# Use of Cyclo-Oxygenase Inhibitors Is Not Associated with Clinical Relapse in Inflammatory Bowel Disease: A Case-Control Study

**DOI:** 10.3390/ph8030512

**Published:** 2015-09-07

**Authors:** Abigail Hensley, Ian L. P. Beales

**Affiliations:** 1 Norwich Medical School, University of East Anglia, Norwich 06360, UK; E-Mail: a.hensley@uea.ac.uk; 2 Department of Gastroenterology, Norfolk and Norwich University Hospital, Norwich 06360, UK

**Keywords:** Crohn’s disease, Ulcerative colitis, cyclo-oxygenase, non-steroidal anti-inflammatory drug

## Abstract

Patients with inflammatory bowel disease (IBD) often have associated conditions, for which anti-inflammatory medication with cyclo-oxygenase (COX) inhibitors may be helpful. The current evidence is conflicting regarding the role of COX-inhibitors in causing relapse in IBD. This case-control study examined the association between the use of COX inhibitors and relapse of IBD. Logistic regression was used to analyse the relationship between COX-inhibitors and IBD relapse. Overall COX inhibitor use (combined non-steroidal anti-inflammatory drugs (NSAIDs) and selective COX-2 agents) had a negative association with relapse of IBD (adjusted OR 0.26, 95% CI 0.09–0.80). This negative association was confined to ulcerative colitis (UC) (adjusted OR = 0.06, 95% CI 0.01–0.50) and no association was found in Crohn’s disease (CD) patients (adjusted OR 1.25, 95% CI 0.18–7.46). The significant negative association between UC relapse and medication use was also seen with non-specific NSAIDs. Selective COX-2 inhibitor use was rare but non-significantly more common in stable patients. There was no association between low-dose aspirin or paracetamol use and relapse of CD or UC. We conclude that COX-inhibitor use was not associated with an increased risk of relapse in UC or CD, and may be protective in UC. Where indicated, NSAIDs should not be withheld from IBD patients.

## 1. Introduction

Inflammatory bowel disease (IBD) is a significant health problem, with a reported incidence of 24.3/100000/year for ulcerative colitis (UC) and 12.7/100000/year for Crohn’s disease (CD) [1]. In 2011, it was thought that there were approximately 240000 patients in the UK with IBD, of which 146000 had UC, 87000 had CD and the remaining 7000 had an unspecified colitis [2].

IBD typically follows a relapsing and remitting pattern, with approximately a third of patients experiencing a relapse each year [3]. The cause of relapse in disease can be due to medication issues, but it has been linked to many different factors, such as smoking, exposure to allergens, infection and in some cases, NSAIDs (non-steroidal anti-inflammatory drugs) [4].

NSAIDs act by inhibiting cyclo-oxygenase (COX) which is the key enzyme by which prostaglandins are formed from arachidonic acid [5]. Prostaglandins are mediators of pain, inflammation and fever; therefore NSAIDs commonly exhibit anti-inflammatory and analgesic properties. COX has two isozymes, COX-1 and COX-2, of which COX-2 is the inducible form mainly responsible for inflammation, whilst the constitutive COX-1 isoform is more involved in a variety of general “household” activities including mucosal defence in the upper GI tract [6].

An association between COX inhibition and relapse in IBD was first noted in case reports over 30 years ago [7]. Since then, there has been considerable research examining the link between NSAIDs and IBD exacerbation. Laboratory studies have shown that the inflamed colon has a significant increase in prostaglandin (PG) E2, PGI2 and thromboxane (TX) A2 [8], caused by an increased expression of COX-2 which is not found in healthy tissues [9]. This increase in prostanoid production might contribute to mucosal inflammation but some of the mediators are thought to contribute to protective mechanism to prevent further damage to the inflamed tissue. The role of many of the fatty acid derived mediators that might improve mucosal inflammation (such a protectins or resolvins) in IBD, and the interactions with NSAIDs has not been sufficiently explored at this point [10].

One theory is that inhibition of prostaglandin production in the colon due to NSAID ingestion causes a disruption in their usual physiological functions, leading to IBD relapse [11]. In the colon, prostaglandins are involved in processes such as mucus production, cell proliferation and regulation of permeability of the epithelial barrier [12]. As these prostaglandins are usually increased in IBD, it is thought that it is this one mechanism which leads to an exacerbation of IBD symptoms. It is now recognised that there is considerable inter-agent variation within the overall class of cyclo-oxygenase inhibitors, not only in the mode of action (aspirin compared to paracetomol compared to traditional non-selective NSAIDS (nsNSAIDs)) but also in non-COX effects (celecoxib appears to inhibit the PI3/Akt kinase pathway and JNK signalling [13,14] and aspirin to inhibit NF-κB signalling [15] and metabolism. Although paracetamol has traditionally been regarded as having minimal peripheral anti-inflammatory actions, it has been reported to be associated with relapse of IBD and it has been postulated that this may be direct toxic effect due to the chemical structure rather than due to COX-inhibition [16,17].

It could be possible that inhibition of COX is beneficial in IBD. As previously mentioned, prostaglandins are potent mediators of inflammation, and COX-2 expression is significantly increased in colonic specimens of active IBD. Experimental models of colitis have shown that taking nimesulide significantly reduced COX-2 expression, neutrophil infiltration and inflammatory oedema [18]. Therefore COX-inhibitors could potentially hasten the resolution of inflammation during a relapse or if taken regularly could prevent exacerbation of symptoms at all. However, currently there are no definitive human studies to support this.

There have been previous epidemiological studies which have shown an association between NSAIDs and IBD relapse [19,20,21], however these have been contradicted by other studies [22,23,24]. This is also true for research focusing on selective COX-2 inhibitors (sCOX-2), with some showing an increase in relapse when taking celecoxib or rofecoxib [25,26] and others showing no effect or even improvement [27,28].

Many of these studies have flawed methodology, have been under-powered, or have not taken in to consideration important confounding factors such as smoking. The case-control studies that have been carried out have had inappropriate control groups (such as irritable bowel syndrome or healthy community controls) or have not been adequately assessed for their NSAID use, as no over the counter medicines have been included, or failed to differentiate between either the types of IBD or types of COX-inhibitor. A systematic review in 2004 concluded the epidemiological evidence was limited and that the association between NSAID use and IBD relapse was weak [29]. A more recent review including several additional studies again concluded that the evidence available is contradictory and confusing, however there is some evidence that selective COX-2 inhibitors are tolerated in the short term [30].

Currently, the information available to clinicians is too perplexing and this has led to many gastroenterologists, general practitioners and specialist nurses advising against using COX inhibitors in IBD patients [22]. However, NSAIDs are a useful medication for IBD patients, as IBD is associated with conditions such as arthralgia, arthritis, sacroiliitis and ankylosing spondylitis, with a reported incidence between 4% and 23% [31]. A study assessing 350 IBD patients found that 36.9% of patients self-reported that they had at least one feature of seronegative spondyloarthritis [32]. These conditions are usually treated with NSAIDs as a first line due to their anti-inflammatory properties, and if it was found that COX inhibitors are safe to use it would benefit these patients greatly.

The aim of this study was to examine an association between COX inhibitors and clinical relapse of IBD, by observing any differences in medication use between those who are stable and those who have relapsed. The secondary aims were to observe if different types of COX inhibitors such as non-selective NSAIDs, selective COX-2 inhibitors, paracetamol and aspirin and their patterns of usage were more likely to cause relapse and if there was any differences between CD and UC.

## 2. Results and Discussion

### 2.1. Characteristics of Study Population

A total of 158 IBD patients were recruited to the study, with 59 patients in relapse and 99 stable controls. 108 patients were recruited from outpatient clinics, 38 from endoscopy appointments and 13 were inpatients. Table 1 shows the demographics. There were slightly more males in the control group (53.5%) compared to the cases (44.1%). The mean age of the stable group (53.3 ± 15.4) was higher than the relapse group (47.6 ± 16.4).

**Table 1:** Characteristics of study population.

	Stable	Relapse	*p* value
Number	99	59	
Male (%) (SD)	53 (53.5)	26 (44.1)	0.25
Mean age, y (SD)	53.3 (15.4)	47.6 (16.4)	0.03
Co-morbidities ^#^ (%)	45 (45.5)	23 (39.0)	0.55
Diabetes * (%)	2 (2.0)	3 (5.1%)	0.37
Smokers or ex-smokers (%)	36 (36.4)	20 (33.9)	0.75
Mean smoking, pack years (SD)	7.2 (13.4)	6.0 (10.8)	0.57
Alcohol drinkers (%)	34 (34.3)	24 (40.7)	0.42
Mean alcohol, units/week † (SD)	5.2 (6.3)	4.5 (6.0)	0.48

Demographic details of subjects in the study; ^#^ Number of subjects with one or more relevant concurrent co-morbidities; * Type 1 or Type 2 diabetes; † Current weekly alcohol consumption.

Almost half of the stable group (45.5%) and 39% of the relapsed group had at least one co-morbidity which was most likely due to the age of the population studied. The percentage of smokers was similar in both groups, and was not statistically different but this was still classed as a confounder due to the known effects of smoking on IBD.

The stable patients had a lower percentage of drinkers than the relapsed group, but on average drank more per week, suggesting that those who did drink alcohol had a higher consumption. However neither of these was significantly different.

### 2.2. Differences in Clinical Presentation

Table 2 shows the clinical features of the IBD experienced by the study participants. In total, 110 patients had UC, 46 had CD and 2 had unspecified colitis. There were a greater proportion of CD patients in the relapsed group (37.2%) compared to the stable group (24.2%), although these differences were not statistically significant. For the purpose of adjustment of odds ratios, the 2 patients with unspecified colitis were classed as missing data.

**Table 2:** Clinical information detailing the nature of inflammatory bowel disease (IBD) in study participants.

	Stable	Relapse	*p* value
Type of IBD, n (%)			
CD	24 (24.2)	22 (37.2)	
UC	73 (73.7)	37 (62.7)	0.14
Unspecified	2 (2.0)	0 (0.0)	
Age at diagnosis, y mean (SD)	40.8 (14.8)	38.0 (16.2)	0.27
Duration of disease, y mean (SD)	12.3 (10.2)	9.6 (8.6)	0.09
Undergone surgery †, n (%)	11 (11.1)	9 (15.3)	0.45
Inpatient admission ‡, n (%)	19 (19.2)	26 (44.1)	0.01

Characteristics of IBD and disease behaviour of subjects in study; Abbreviations: CD = Crohn’s disease, UC = Ulcerative colitis; † Patient had undergone surgery directly due to inflammatory bowel disease at some time during the disease course; ‡ Patient had been admitted to hospital directly due to inflammatory bowel disease at some time during the disease course.

Previous hospital admission rates for IBD differed between the cases and controls, 44.1% of those in relapse had inpatient stays due to their IBD, compared to 19.2% of those who were stable. Features such as age at diagnosis, duration of time since diagnosis and number of patients who had undergone surgery for their IBD did not differ significantly between the two groups.

### 2.3. Medications for IBD Control

Table 3 shows medications used by the study population for IBD symptom control over the prior 3 months. Most patients were taking 5-aminosalicylate drugs (5-ASAs, mesalazines) with 69.7% of stable patients taking them long term. There was a statistically significant difference between stable and relapse patients in terms of 5-ASA use, with a *p*-value of 0.02 and OR of 0.45 (95% CI 0.23–0.88). This is probably directly explained by the lower overall use of 5-ASAs by Crohn’s patients (Twenty two (47.8%) CD patients were taking 5-ASAs compared to 76 (69.1%) of UC patients, which was also a statistically significant difference (*p* = 0.01, OR = 2.44, 95% CI 1.20–4.99).

**Table 3:** Medications taken for IBD.

	Stable, n (%)	Relapse, n (%)	*p* value	OR (95% CI)
5-ASAs *	69 (69.7)	30 (50.8)	0.012	0.45 (0.23–0.88)
UC	63	26	0.053	0.38 (0.14–1.02)
CD	6	4	0.60	0.67 (0.15–2.87)
Steroids †	1 (1.0)	15 (25.4)	<0.001	33.41 (4.28–260)
UC	1	7	<0.001	16.4 (2.4–386)
CD	0	8	-	-
Azathioprine, MP or MTX	32 (32.3)	12 (20.3)	0.10	0.52 (0.23–1.11)
UC	18	6	0.32	0.59 (0.19–1.62)
CD	14	6	0.04	0.27 (0.09–0.94)
Anti TNF-α drugs	3 (3.0)	3 (5.1)	0.67	1.71 (0.34–8.78)
UC	1	0	-	-
CD	2	3	0.33	1.55 (0.21–14.2)

Medications taken for IBD over the past 3 months; Abbreviations: 5-ASAs = 5-aminosalicylic acid, MP = mercaptopurine, MTX = methotrexate; * Includes mesalazine, sulphasalazine, balsalazide and olsalazine preparations; † oral and IV corticosteroids (prednisolone, methylprednisolone, budesonide, beclomethasone).

Azathioprine, mercaptopurine and methotrexate use was considered as one single variable. Their use was 12% higher in the stable group of patients (32.3% compared to 20.3%). Although the difference was not statistically different (*p* = 0.10, OR = 0.54, 95% CI 0.25–1.14), it was treated for as a potential confounder along with 5-ASAs and used for adjustment of odds ratios in Table 4.

### 2.4. Use of COX-Inhibitors

There were 34 patients in total who had used either selective COX-2 inhibitors or nsNSAIDs, accounting for 21.5% of the whole study population. Of these, 25 were stable and 9 were in relapse. Table 4 shows there was a significant negative association found between the use of COX-inhibitor (NSAID and COX-2 inhibitors combined) and IBD relapse, with an adjusted odds ratio of 0.26 with a 95% confidence interval of 0.09–0.80, suggesting a potentially protective effect (adjusted for age; sex; type of IBD; smoking; 5-ASA, steroid and immunomouldator use).

**Table 4:** Medication use in inflammatory bowel disease patients in the three months prior to assessment.

	Stable (%)	Relapse (%)	*p* value	Unadjusted OR (95%CI)	Adjusted OR (95% CI) ^#^
All NSAIDs ^¥^	25 (25.3)	9 (15.3)	0.14	0.53 (0.23–1.24)	0.26 (0.09–0.80)
Non-selective NSAID	19 (19.2)	8 (13.6)	0.36	0.66 (0.27–1.62)	0.42 (0.14–1.24)
Selective COX-2 inhibitor	6 (6.1)	1 (1.7)	0.26	0.27 (0.03–2.28)	**
Paracetamol	37 (37.4)	28 (47.5)	0.21	1.51 (0.79–2.91)	1.43 (0.65–3.14)
Aspirin	11 (11.1)	3 (5.1)	0.20	0.43 (0.16–1.60)	0.95 (0.21–4.25)

Medication use in inflammatory bowel disease patients in the 3 months prior to assessment; Abbreviations in Table 4: OR- odds ratio, CI—confidence interval; COX-cyclooxygenase, NSAID—non-steroidal anti-inflammatory drug; ^#^ Odds ratios adjusted for age; sex; type of IBD; smoking, previous admission, duration of IBD or surgery and use of 5-aminosalicylates, azathioprine, mercaptopurine, methotrexate and steroids; ^¥^ This category combined usage of NSAIDs and COX-2 inhibitors; ** Values too small to calculate adjusted odds ratio.

Selective COX-2 inhibitors and nsNSAIDs classes were also examined individually. In total 27 patients were taking nsNSAIDs, 19 in the stable group and 8 who had relapsed. The difference between groups was also not statistically significant with a *p* value of 0.36 and adjusted odds ratio of 0.42 (95% CI 0.14–1.24). The type of nsNSAID most often used was ibuprofen (21/27: 77.8%) (there was less frequent use of naproxen, diclofenac, indomethacin or mefenamic acid) and the type of NSAID did not differ significantly between cases and controls (*p* = 1.00). In all cases standard therapeutic doses were taken, no subject reported taking non-standard doses.

Only seven patients in total were taking selective COX-2 inhibitors, five of these were using celecoxib and two etoricoxib. Of the seven patients, six had stable disease and one was in relapse. This was not a significant difference, although the number studied was small (*p* = 0.26, OR 0.27 (95% CI 0.03–2.28)).

Paracetamol use was examined separately and was associated with non-significant increase in IBD relapse rates (unadjusted OR 1.51 (95% CI 0.79–2.91)), (adjusted OR 1.43 (95% CI 0.65–3.14). Out of the 65 patients taking paracetamol in the whole study population, six of these were taking it on prescription and 59 had bought it over the counter. This did not differ between stable and relapsed patients. Similarly there was no significant association between use of paracetamol and relapse in either Crohn’s disease (OR 1.27 (95% CI 0.54–3.65), adjusted OR 1.33 (95% CI 0.48–3.93)) or ulcerative colitis, (OR 1.67 (95% CI 0.48–3.39), adjusted OR 1.73 (95% CI 0.43–3.97)) although the numbers in each group are relatively small and the resulting confidence intervals relatively wide. The data on paracetamol are possibly more open to bias by indication, it is possible that subjects experiencing abdominal pain due to their IBD before experiencing a more obvious flare up may take paracetamol for symptomatic treatment but may avoid aspirin and NSAIDs in this situation.

Aspirin was used by 11 (11.1%) stable patients compared to 3 (5.1%) relapsed patients, as seen in Table 4. The difference between the groups was not statistically significant (*p* = 0.20, unadjusted OR 0.43 (95% CI 0.16–1.60), adjusted OR 0.95 (95% CI 0.21–4.25)). Ten of these patients were taking aspirin at the dose of 75 mg once daily for prevention of cardiovascular disease, the other patient had taken a dose of 300 mg four times daily for a period of 12 days.

Overall COX inhibitor use and NSAID use was additionally analysed by type of IBD. These analyses are shown in Table 5 and Table 6. Relapse of CD did not show any association with either overall COX inhibitor use or nsNSAID use with *p* values of 0.51 and 0.73 respectively and odds ratios of 1.25 (95% CI 0.18–7.46) and 1.15 (95% CI 0.18–7.46) respectively. However, there was a significant inverse association with overall COX inhibitor use and relapse of UC, with a *p* value of 0.01 and adjusted odds ratio of 0.06 (95% CI 0.01–0.50). This suggests that COX-inhibitors might have a protective effect in UC. Furthermore, analysis on nsNSAIDs separately also showed a similar statistically significant negative association between relapse in UC and nsNSAID use when adjusted for confounders (*p* = 0.08, adjusted OR = 0.16, 95% CI 0.03–0.97) (Table 6). Selective COX-2 inhibitors were not analysed by type of IBD due to the small number of patients taking these medications. There was no significant association either positive or negative between use of all COX-inhibitors, aspirin or paracetomol or nsNSAIDs and relapse in Crohn’s disease.

**Table 5:** Overall COX inhibitor (nsNSAID and/or sCOX-2 inhibitor) use in the past 3 months in IBD patients categorised by type of disease.

Type of IBD	Stable (%)	Relapse (%)	*p* value	Unadjusted OR (95%CI)	Adjusted OR (95% CI) #
CD	5 (20.8)	7 (31.8)	0.51	1.77 (0.47–6.72)	1.25 (0.18–7.46)
UC	20 (27.4)	2 (5.4)	0.01	0.15 (0.03–0.67)	0.06 (0.01–0.50)

Medication use in inflammatory bowel disease patients in the 3 months prior to assessment; Percentages given are for % of patients within type of IBD. Abbreviations in Table 5: OR- odds ratio, CI—confidence interval; COX-cyclooxygenase, NSAID—non-steroidal anti-inflammatory drug; ^#^ Odds ratios adjusted for age; sex; type of IBD; smoking, previous admission, duration of IBD or surgery and use of 5-aminosalicylates, azathioprine, mercaptopurine, methotrexate and steroids; ^¥^ This category combined usage of NSAIDs and COX-2 inhibitors.

**Table 6:** Non-specific NSAID use in the past 3 months in IBD patients categorised by type of disease.

Type of IBD	Stable (%)	Relapse (%)	*p* value	Unadjusted OR (95%CI)	Adjusted OR (95% CI) #
CD	5 (20.8)	6 (27.3)	0.73	1.43 (0.37–5.56)	1.15 (0.18–7.46)
UC	14 (19.2)	2 (5.4)	0.08	0.24 (0.05–1.12)	0.16 (0.03–0.97)

Medication use in inflammatory bowel disease patients in the 3 months prior to assessment; Percentages given are for % of patients within type of IBD. Abbreviations in Table 6: OR- odds ratio, CI—confidence interval; COX-cyclooxygenase, NSAID—non-steroidal anti-inflammatory drug; ^#^ Odds ratios adjusted for age; sex; type of IBD; smoking, previous admission, duration of IBD or surgery and use of 5-aminosalicylates, azathioprine, mercaptopurine, methotrexate and steroids; ^¥^ This category combined usage of NSAIDs and COX-2 inhibitors.

The aim of the current study was to explore the use of cyclo-oxygenase inhibitors and relapse of IBD: use COX-inhibitors for all indications was included. The majority of the COX-inhibitor use (80%) was for musculosketal indications, with headache and menorrhagia contributing the remainder, it is possible that the underlying indication for anti-inflammatories affects the relationship with IBD but a larger study would be required to explore such sub-group analysis.

Despite the well know effects on the upper GI tract acid-suppressive drugs were uncommonly taken in this cohort (stable 12/99, relapse 6/59), whilst it remains possible that acid-suppressive drugs, co-prescribed with COX-inhibitors, leading to changes in the GI microbiome may influence relapse of IBD, our study shows no such effect, albeit with small numbers.

In order to capture all use of COX-inhibitors, we have used broad definition of drug-exposure that encompasses both occasional, intermittent use and regular, long-term daily dosing. Although our data overall do not show a link between use of COX-inhibitors and relapse of IBD, further larger studies may be required to explore different drugs and dose regimens.

## 3. Experimental Section

### 3.1. Study Design

This study was designed as a retrospective case-control study based on a previously used methodology [33,34].

### 3.2. Subject Population

The study participants were recruited from patients attending the Gastroenterology department at the Norfolk and Norwich University Hospital for their standard IBD care from October 2012 to July 2013. This included inpatient admissions, endoscopy appointments and routine or urgent outpatient clinics. All patients were over 18, had a confirmed diagnosis of IBD and spoke fluent English. Management of the patients’ IBD remained the responsibility of the relevant gastroenterologist and was independent of the study. Those who were outpatients and had relapsed in the last 3 months but treatment escalation had led to improvement in symptoms by the time of review, or those that had a flare in symptoms that had settled by the time of review were excluded from the study to avoid recall bias.

All subjects gave informed consent and the project was approved by the Norfolk and Norwich University Hospital Trust Research Governance Committee and the Norfolk Research Ethics Committee.

Information on demographics and possible exposures including prescribed and over the counter drugs and current disease activity was obtained from subjects via a structured interview with a trained student researcher (ALH), prescribed drug exposure was cross-referenced with clinical records. The structured interviews took place in a private room at the time of the planned outpatient endoscopy or clinic appointment or at a mutually convenient time for those patients seen as inpatients. Additional scoring of available endoscopy appearances was performed independently of decisions on clinical management and drug exposure (ILB).

Cases: The participants who were inpatients or outpatients in clinical relapse when seen were defined as cases. Clinical scoring using the Harvey-Bradshaw index (HBI) or Simple Colitis Activity Index (SCAI) was used to define relapse. These tools have both been validated to determine clinical relapse in IBD patients [35,36,37].

A score of greater than 3 using either the HBI or the SCAI was defined as a relapse. Relevant endoscopic data were not available for all subjects (as is typical in everyday practice) but where available these were scored according to the Endoscopic Mayo score for ulcerative colitis [38] and The Simple Endoscopic Score for Crohn’s Disease (SE-CD) endoscopic score for Crohn’s disease [39]. Relapse was defined as an endoscopic Mayo score of 2 or above and Crohn’s score of 2 or above in any ileoccolonic segment. When endoscopic and clinical classification of relapse differed, the endoscopic scoring was used.

Controls: Controls were defined as outpatients in clinical remission for greater than 3 months at the time of review.

Drug exposure: The use of NSAIDs, selective COX-2 inhibitors, aspirin, paracetamol as well as other prescribed (including those for IBD) and non-prescribed drugs, over the past 3 months were recorded for each patient. For all drugs, dosage, frequency and duration of use were noted. For this study any use of a COX-inhibitor in the preceding 3 months was regarded as positive exposure. Information about potentially confounding factors including alcohol consumption and tobacco smoking [40] and the relevant IBD history (type of IBD, duration of disease, previous surgery and admission), was also collected.

### 3.3. Sample Size Estimation

The project aimed to recruit 224 subjects. Assuming a prevalence of NSAID use of 10% in stable patients, a sample size of 56 relapsed patients and 168 controls was planned to have 80% power to detect a significant association (defined as an odds ratio of 3.0) between relapse and NSAID use.

### 3.4. Statistical Analysis

All analyses took place using SPSS version 18.0. Continuous variables were compared using the unpaired two sample *t*-test and categorical variables were compared using the chi-squared test or Fisher’s Exact test if any of the expected counts in the calculation were less than 5. The percentage of patients that used COX-inhibitors (this group included NSAIDs and selective COX-2 inhibitors combined), selective COX-2 inhibitors, NSAIDs, paracetamol, aspirin in the past 3 months in the case group and control group was compared using the chi-squared test (or Fisher’s Exact test). Odds ratios with 95% confidence intervals were calculated to quantify the difference and statistical significance was set at a *p* value of 0.05. Odds ratios were then adjusted using logistic regression for any confounders.

It was pre-determined that sex, type of IBD, smoking and use of steroids, 5-ASAs or current use of immunodulator therapies would treated as confounders as these all have an effect on relapse rates. Any other variable found to be statistically significant was also adjusted for. It was pre-planned to perform subgroup analysis of CD and UC and to examine both overall COX-inhibitor use (nsNSAIDs and sCOX-2 inhibitors) and overall NSAID use.

## 4. Conclusions

In conclusion, this case control study shows that COX- inhibitor use (nsNSAIDS and COX-2 selective agents) was not associated with an increased risk of relapse of inflammatory bowel disease. In fact nsNSAIDs and COX-2 selective agents were significantly inversely associated with relapse of UC but not CD. This suggests that COX-inhibitors may have a protective effect against relapse and maintain remission of UC. There was no statistically significant relationship either positive or negative between NSAID-use and relapse of CD, although the confidence intervals were relatively wide. Neither aspirin nor paracetamol was associated with either increased or decreased relapse rate in IBD, again with relatively wide-confidence intervals. It is recommended that further work needs to be carried out to repeat and expand these observations in larger populations and to investigate the potential biological mechanisms and the differences between UC and CD, as well as differences between drugs and COX-inhibitor classes would be beneficial. At this stage, as there was no evidence of a harmful effect of COX-inhibitors on relapse of IBD, we do not feel that COX-inhibitors, when indicated by other symptoms, should be withheld from patients with particularly ulcerative colitis, but also Crohn’s disease.

## Abbreviations

COX: cyclo-oxygenase
CD: Crohn’s disease
UC: ulcerative colitis
IBD: inflammatory bowel disease
HBI: Harvey-Bradshaw index
SCAI: Simple Colitis Activity Index
NSAID: non-steroidal anti-inflammatory drug
nsNSAID: non-selective non-steroidal anti-inflammatory drug
PG: prostaglandin
sCOX-2: selective cyclo-oxygenase-2 inhibitor
OR: odds ratio
